# Real-World Experience Using Bimekizumab for the Treatment of Moderate-to-Severe Psoriasis From a Single Outpatient Dermatology Clinic

**DOI:** 10.1177/12034754231217205

**Published:** 2023-12-29

**Authors:** Katrina D. Cirone, Fiona E. Lovegrove

**Affiliations:** 1 Schulich School of Medicine and Dentistry, Western University, London, ON, Canada; 2 Lovegrove Dermatology, London, ON, Canada

**Keywords:** bimekizumab, psoriasis, biological therapy, real-world data, systemic therapy

To the Editor,

Bimekizumab, a humanized monoclonal immunoglobulin G1 antibody that selectively inhibits both interleukin (IL)-17A and IL-17F, is capable of achieving high rates of skin clearance in adults with moderate-to-severe plaque psoriasis when dosed every 4 or 8 weeks.^1-5^ Four large, randomized, multinational, placebo and/or active comparator-controlled phase III clinical trials, BE VIVID, BE READY, BE SURE, and BE RADIANT have demonstrated bimekizumab is highly effective, well-tolerated, and safe in adults with moderate-to-severe psoriasis.^1-4^ Adverse events reported in these trials included upper respiratory tract infections (most frequently nasopharyngitis), oral candidiasis, headaches, and injection site reactions. Due to the recent approval of bimekizumab for treatment of moderate-to-severe psoriasis, real-world efficacy and safety data is currently limited. The purpose of this retrospective chart analysis was to identify real-life outcomes of bimekizumab usage in adults with moderate-to-severe psoriasis.

A retrospective chart review of 20 patients with moderate-to-severe psoriasis who were prescribed bimekizumab treatment at a community dermatology office in Ontario, Canada was conducted. For inclusion in this review, patients required a diagnosis of moderate-to-severe psoriasis, defined as Body Surface Area ≥10%, Psoriasis Area and Severity Index (PASI) ≥10, or with special site involvement (face, genitals, hands, or feet), and were prescribed bimekizumab. Bimekizumab was prescribed according to product monograph and the dosing interval was optimized by the physician with the frequency increased to up to 320 mg every 4 weeks as needed, based on clinical response.^5^ Clinical efficacy of bimekizumab was evaluated using the PASI scores at baseline and at 4 to 6 months after bimekizumab initiation. Patient safety was measured based on the presence of any adverse events during bimekizumab treatment.

Of the 20 psoriasis patients managed on bimekizumab, 11 had follow-up data at the time of review, and 1 patient discontinued treatment. Patient demographics and baseline clinical characteristics of the entire cohort are presented in Supplementary Table S1 and treatment characteristics are presented in Supplementary Table S2. In our cohort, all patients included in the analysis achieved clinically significant clearance of psoriasis following treatment with bimekizumab, and a 93.7% reduction in PASI score was seen at follow-up (Supplementary Table S3 and Figure S1). Bimekizumab was well-tolerated overall with no serious adverse events seen, with only 3 patients (18.8%) developing oral candidiasis.

This is the first study assessing real-world usage and effectiveness of bimekizumab in Canadian patients with moderate-to-severe psoriasis. This analysis showed bimekizumab used in clinical practice with patients maintained on treatment every 8 weeks, is effective psoriasis management, regardless of a patients’ previous experience with biologics. Optimization through increased treatment frequency resulted in significant disease improvement and was not associated with any novel safety concerns. Future analyses with larger populations and longer follow-up should continue to be reported to add to our knowledge of bimekizumab usage in a clinical setting. Overall, our analysis indicated that the results reported by randomized controlled clinical trials translate well into real-world clinical practice, and demonstrate the efficacy and safety of bimekizumab in treating psoriasis.

## Supplemental Material

sj-docx-1-cms-10.1177_12034754231217205 – Supplemental material for Real-World Experience Using Bimekizumab for the Treatment of Moderate-to-Severe Psoriasis From a Single Outpatient Dermatology Clinic Supplemental material, sj-docx-1-cms-10.1177_12034754231217205 for Real-World Experience Using Bimekizumab for the Treatment of Moderate-to-Severe Psoriasis From a Single Outpatient Dermatology Clinic by Katrina D. Cirone and Fiona E. Lovegrove in Journal of Cutaneous Medicine and Surgery
