# Supplementary material for: Real-World Experience Using Bimekizumab for the Treatment of Moderate-to-Severe Psoriasis From a Single Outpatient Dermatology Clinic
Source: J Cutan Med Surg. 2023 Dec 29;28(1):78–9. doi: 10.1177/12034754231217205 (PMC10908189; doi:10.1177/12034754231217205)

**Supplementary Material**

**Real-world Experience using Bimekizumab for the Treatment of Moderate-to-Severe Psoriasis from a Single Outpatient Dermatology Clinic**

Katrina D. Cirone HBSc and Fiona E. Lovegrove MD PhD FRCPC

**Table of Contents**

Supplementary Table S1. Baseline Patient Characteristics (N = 20)

Supplementary Table S2. Treatment Characteristics

Supplementary Table S3. Clinical outcomes following treatment with bimekizumab

Supplementary Figure S1. Baseline and follow-up PASI scores

**Supplementary Table S1.** Baseline Patient Characteristics (N = 20)

| Variable | Value |
| --- | --- |
| Age (years), mean + SD | 50.5 + 13.0 |
| Female, n (%)  Special site involvement, n (%)  Any  Hands  Feet  Face  Genitals  Comorbidities, n (%)  Any  Psoriatic Arthritis  Localized Pustular Disease  Hidradenitis Suppurativa  Prior systemic therapy (conventional and/or biologic), n (%)  Prior conventional systemic therapy, n (%)  Prior biologic therapy, n (%)  No. of previously failed systemic therapies, mean + SD  Conventional systemic therapies prior to bimekizumab, n (%)  Methotrexate  Acitretin  Alitretinoin  Apremilast  Biologic therapies prior to bimekizumab, n (%)  Secukinumab  Risankizumab  Ixekizumab  Adalimumab  Guselkumab  Brodalumab  Golimumab  Previously failed biologic therapies, n (%)  Biologic-naïve  1 biologic  > 2 biologics | 9 (45.0)  20 (100.0)  15 (75.0)  14 (70.0)  3 (15.0)  3 (15.0)  13 (65.0)  8 (40.0)  6 (30.0)  1 (5.0)  19 (95.0)  17 (85.0)  10 (50.0)  2.4 + 1.6  13 (65.0)  7 (35.0)  3 (15.0)  3 (15.0)  5 (25.0)  4 (20.0)  3 (15.0)  2 (10.0)  2 (10.0)  1 (5.0)  1 (5.0)  10 (50.0)  4 (20.0)  6 (30.0) |

Abbreviations: *N*, number of individuals; SD, standard deviation.

**Table S2.** Treatment Characteristics

| Variable | Value |
| --- | --- |
| Treatment Duration (months), mean + SD [range]  Dosing Interval, n (%)  Every 8 weeks  Increased frequency to every 4 weeks | 6.9 + 3.1 [1.5,13.5]  11 (91.7)  1 (8.3 |

Abbreviations: SD, standard deviation.

**Supplementary Table S3.** Clinical outcomes following treatment with bimekizumab

| PASI, mean + SD [range] (n)  Baseline  4 to 6 month follow-up  PASI improvement at 4 to 6 month follow-up, n (%)  PASI 90  PASI 100  PASI Relative reduction (%)  Discontinuation, n (%)  Adverse events  Oral candidiasis, n (%) | 9.1 + 5.1 [4.0-18.6] (15)  0.6 + 0.9 [0.0-2.4] (9)  7 (77.8)  5 (55.6)  93.7  1 (6.3%)  3 (18.8) |
| --- | --- |

Abbreviations: *N*, number of individuals; SD, standard deviation; PASI, Psoriasis Area and Severity Index.

**Supplementary Figure S1.** Baseline and follow-up PASI scores


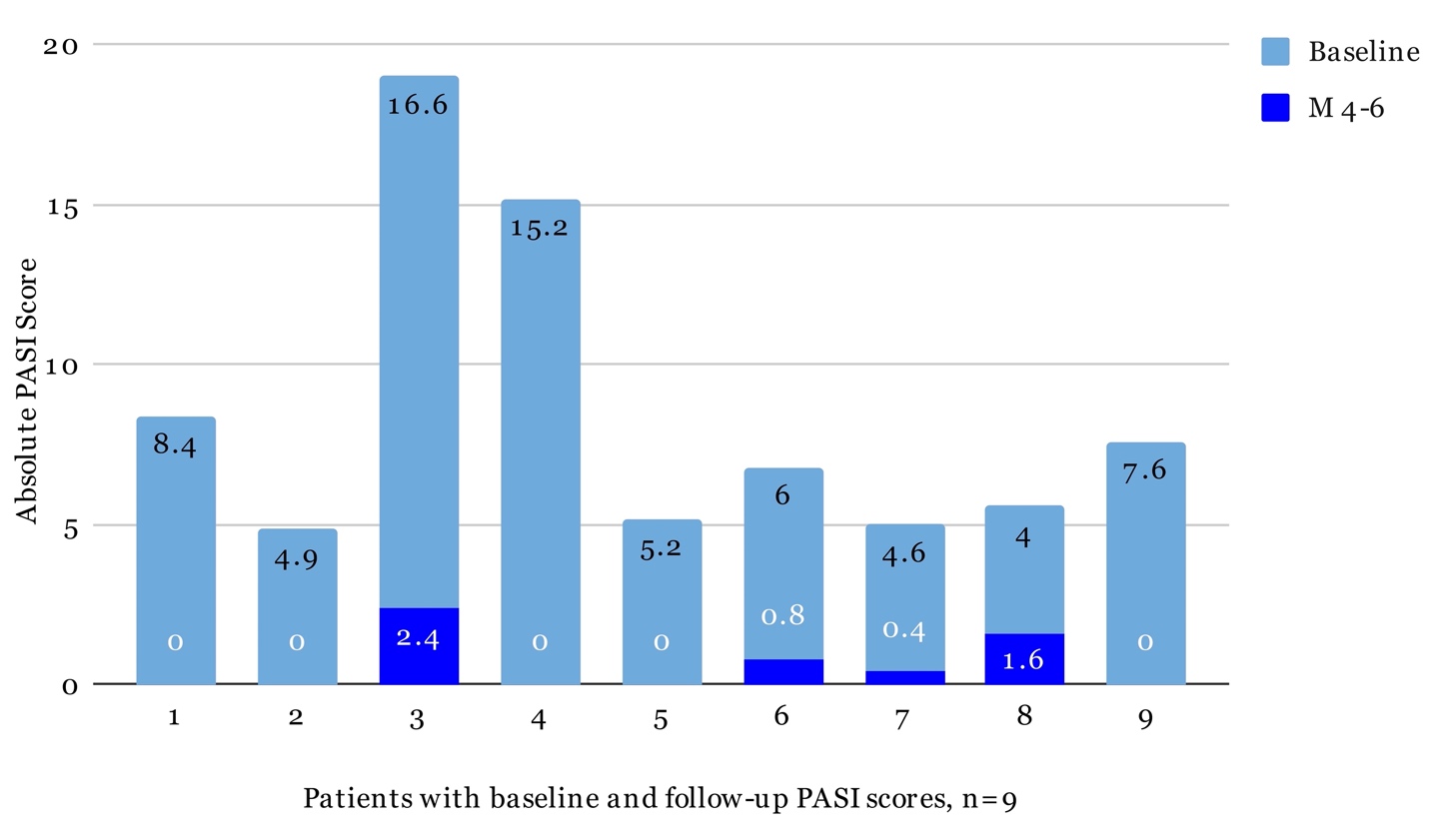

Supplement: sj-docx-1-cms-10.1177_12034754231217205 – Supplemental material for Real-World Experience Using Bimekizumab for the Treatment of Moderate-to-Severe Psoriasis From a Single Outpatient Dermatology Clinic [file sj-docx-1-cms-10.1177_12034754231217205.docx]
